# Exploring the Challenges and Opportunities for Female Medical Students Engaged in Research in Saudi Arabia: A Qualitative Study

**DOI:** 10.7759/cureus.43607

**Published:** 2023-08-16

**Authors:** Emily Wilson, Reem Elmokattaf, Roaa Aljumaa, Ghada Almasri, Maryam T Altayeb, Muhammad Sajid

**Affiliations:** 1 English Department, Alfaisal University, Riyadh, SAU; 2 College of Medicine, Alfaisal University, Riyadh, SAU; 3 Pathology Department, Alfaisal University, Riyadh, SAU

**Keywords:** research, saudi arabia, student perception, feminist methodology, qualitative study, gender, women

## Abstract

While researchers have made great strides in expanding opportunities for women in medical research, some gender imbalance persists, particularly in the context of the Arab world. The purpose of our study was to discover obstacles that female medical students have faced in conducting research. We conducted our study at a small private university in Riyadh, Saudi Arabia, and we used a qualitative, feminist methodology. In April 2022, we collected data from four group interviews with 21 female undergraduate medical students who had experience participating in research projects. The study’s aim was to elicit participants' perspectives on the barriers women face when conducting medical research through all phases of the research process, including expressing interest in a particular field, finding faculty support, performing research activities, and assessing research outcomes. The inductive thematic framework data analysis revealed three major themes expressed by participants: differing expectations for female students versus male students in conducting research; challenges for female students in securing research opportunities; and practical challenges for female students in conducting research. Findings from this study suggest that targeted interventions such as mentorship programs can help female students overcome obstacles and work toward equal participation of female and male students in medical research.

## Introduction

In the last few years, members of the medical community have expressed strong interest in supporting the participation of women in scientific research. Some researchers have made changes in research practice and design to increase the representation of women [1]. However, despite recent advances, gender imbalance persists in many scientific fields, including medicine. In some Middle Eastern and Arab countries where commitment to gender equality and female empowerment is still emerging, cultural expectations can provide another layer of complication when it comes to women’s participation in research. For instance, a study from Qatar found that there were fewer female researchers than male researchers, and teams involving women published fewer articles [2]. Moreover, students’ development of positive attitudes toward scientific research is a fundamental element of modern undergraduate medical education curricula [3]. A 2019 study showed that graduate male students had unquestionably positive attitudes toward research in comparison to female graduate students [4]. On the other hand, a study conducted in Saudi Arabia showed that female undergraduate medical students with previous research experience had more positive attitudes toward undergraduate research [5].

Our literature review revealed several studies that explore the involvement of female undergraduate medical students in research and the obstacles these students may face. In one study from Portugal, authors concluded that male medical students are more likely than female students to be involved in research [6]. The gender gap could be due to sociocultural barriers (e.g., lack of female role models and decreased levels of autonomy). Female students also tend to focus more on academic performance, which may decrease their engagement in undergraduate research [6]. A study from Saudi Arabia that investigated attitudes toward health research among male and female medical students revealed that female students had a positive attitude toward research. However, men made more contributions to research than women did [7]. Another study from Saudi Arabia found that more male medical students than female medical students engaged in undergraduate research. It also explored obstacles to research participation among all medical students, including lack of interest, lack of research opportunities, and socio-cultural barriers [8].

One study from Saudi Arabia showed that in contrast to female medical students, male medical students were becoming increasingly involved in undergraduate research [9]. Additionally, this study mentioned barriers perceived by female medical students in Saudi Arabia toward careers in academic medicine. These barriers included a lack of female role models and the presence of competing pressures, including teaching, clinical duties, and family obligations [10]. On the other hand, one study from Abha, Saudi Arabia, suggested that there was no relationship between gender and barriers to conducting medical research in Saudi Arabia [11].

Given the lack of specific focus on the obstacles faced by undergraduate women in medical research, our purpose was to use qualitative methods to explore these students’ perspectives on research opportunities and challenges in Saudi Arabia. We hope that our research might suggest ways to increase female undergraduate students’ involvement in research.

This article was presented in the 14th Annual Student Poster Competition at Alfaisal University on March 2, 2023, and submitted as an abstract in the AMEE Glasgow 2023 Conference on August 26 to 28, 2023. It was also previously posted to the Research Square preprint server on May 19, 2023.

## Materials and methods

We employed a qualitative case study design to explore the female students’ perspectives toward research opportunities in the College of Medicine at Alfaisal University in Riyadh. This study consisted of four group interviews conducted in April 2022. We obtained approval for this study from the university’s institutional review board (IRB-20153).

We employed feminist methodologies by centering the lived experiences of women, analyzing those experiences in light of socially constructed gender norms, and approaching our participants as producers of knowledge [12]. This approach gave us a framework for constructing knowledge based on lived experience, which we then used as the basis for an expanded understanding of the systems in which female undergraduate students operate.

The method of data collection for this study was a series of four focus groups. Our sampling technique was purposeful sampling [13]. The study participants were female medical students at Alfaisal University who were involved in research and were willing to participate in a group interview. Because our primary research interest was women’s perspectives, male students were excluded from the study. Additionally, female students who were not involved in any research projects were excluded. A list of female students was compiled using the research office’s database. We then sent an email (that included the research objectives) to these students asking them to participate in the study. Once students had volunteered to participate in the study, we interviewed four to six students at a time. A total of four hour-long interviews were conducted in English, led by a principal investigator (EMW) with experience in conducting qualitative interviews. Twenty-one female medical students were interviewed for this study: six second-year students, 13 third-year students, one fourth-year student, and one fifth-year student. Interviews were conducted until we reached thematic saturation.

We created an interview guide that featured semi-structured, open-ended questions that allowed participants to express different perspectives and allowed our team to collect in-depth data. After we conducted the first interview, we transcribed and analyzed the responses. Based on our analysis, we made minor changes to the interview guide to clarify our meaning and obtain more useful information. The interaction during interviews featured a dynamic exchange of ideas, with free-flowing discussions that addressed different aspects of the issues. The interviewers asked all questions in the same general sequence. To maintain confidentiality, transcripts were anonymized, and access to all files was limited to PI and co-PIs.

The method of data analysis used for this study was an inductive thematic framework analysis, which enabled us to categorize conversational text and identify patterns. Repeated reviews of the transcripts allowed us to identify codes and discover relationships between ideas and codes, and we continued the process of coding until data saturation was reached [14-16].

The data analysis phase also involved generating a series of descriptive and in vivo codes [15]. We identified a total of 19 codes from the four interviews. The components of every code included the origin, definition, and the best examples that were mentioned by the participants. Disagreements about coding were resolved by group consensus, and codes were compared to ensure inter-coder reliability. Two out of the 19 codes contained counterexamples. We then narrowed down the codes to nine that were the most consistent with the study aims. We generated descriptive memos from those nine codes to analyze our data effectively. The memos included a brief description of our analysis of these codes as well as examples taken from the transcribed interviews. From these memos emerged the three main themes discussed in the results section.

## Results

In our analysis of participants’ perspectives on the challenges women face in conducting research, three major themes surfaced: differing expectations for female students versus male students in conducting research, challenges for female students in securing research opportunities, and practical challenges for female students in conducting research.

**Table 1:** Findings from thematic analysis

Theme	Key findings
Differing expectations for female versus male students in conducting research	Students expressed that there were different expectations regarding female students' abilities
	Expressions of surprise from faculty regarding results achieved by female students
Challenges for female students in securing research opportunities	Specific challenges in securing research opportunities and approaching male faculty to create meaningful research
	Social and cultural barriers: "shyness" due to their own sensitivity, fear of bothering busy faculty, gender barriers, and feeling undeserving of opportunities
Practical challenges for female students in conducting research	Lack of opportunities related to transportation issues
	Compromised sense of safety while performing tasks in male-dominated spaces

Theme 1: differing expectations for female students versus male students in conducting research

Some participants reported that faculty seemed to indicate differing expectations for female students versus male students. These differing expectations surfaced in various ways throughout the research process, from gauging interests to assuming abilities to judging outcomes.

Some participants reported different expectations regarding female students’ abilities. In discussing interactions with faculty, one participant remarked, “Even if you think that I cannot do this, I will proceed to do that specific thing…just to try to prove otherwise, [that] we can do whatever males can do.” Other participants commented on their need to outperform male colleagues to demonstrate their abilities: “Personally, always I think I should do better than a male does. Because we [are] always in a circle when we need to prove that…we deserve this opportunity...we deserve this thing that we had.” One participant linked women’s need to prove themselves to a broader historical context. She noted that women are “relatively newer to the workforce…compared to our ancestors who were mostly housewives at home,” and described the effects of this historical shift: “You need to prove yourself if you're here, if you're going to be paid as much as [a man].”

Other participants described the surprise that some faculty members expressed at the results that the female students were able to achieve. Some participants interpreted over-praise as a signal that female students were not expected to perform as well as their male counterparts:

Participant: Sometimes I feel like they look at you like they don't expect a lot and then when you do something they’re impressed more than if it was a male.

Interviewer: Can you give a specific example?

Participant: So for example, let's say…the poster day. I feel like sometimes when they see that it's [a] group of females…they would be more impressed only because we are females. So, they understand that females can do a lot and they understand that yes, we're going to do it. But when they actually see us doing something great, they would be more impressed.

Similarly, another participant interpreted faculty members’ over-praise as indicating low expectations. She commented: “I think at the beginning we think that [praise] is something nice…when we think about it more…it’s only because they don't expect that much…when they praise you, they're not trying to belittle you, but they're actually genuinely impressed because in their minds they did not think, for example, that yes, you can do it…maybe you're praising me only because I'm a female and you did not expect me to do this.”

The point where the participant noted that praise started to feel like low expectations was when she compared it to praise that a male student might receive for the same work: “I did not start to feel happy when someone would praise me for these little things that I don't think other male [students] would get as much praise from it.”

One female student voiced dissent after listening to some of the above comments. She said, “I personally have a different opinion…I know a professor who worked with both male and female students from the university, and they were impressed from both the male and the females…and I think I don’t know if we really have enough evidence to jump into this conclusion…[that] they’re belittling me because I’m a female and they don’t expect this from me.” While she acknowledged the possibility of the situation her colleagues described, she expressed hesitation about jumping to the conclusion that low expectations for a student were tied to gender.

Some participants who perceived lower expectations said they were determined to prove those expectations wrong. As one participant noted, “I understand why they are praising me in that way…and honestly personally I get more competitive…Like it really pushes me forward.” On the other hand, as we will see in the next section, other participants responded with hesitation and fear of rejection.

Theme 2: challenges for female students in securing research opportunities

Our participants expressed specific challenges in securing research opportunities and approaching faculty to create meaningful research. Some participants reported difficulty in finding equal opportunities to conduct research in situations where professors seemed to take male students more seriously. For example, one interviewee mentioned that faculty turned her down while giving similar opportunities to a male student within the same time frame. “But I feel like, indirectly…they would say, oh, we don't have any projects going on, but then we see that male students are joining them.”

As one participant stated, “Sometimes in research you get a lot more doors shut in your face than doors open.” There was a consistent emphasis on the frequency of rejection, which happened to many students, regardless of gender. However, one female student reported multiple rejections when trying to propose a research idea, when the same doctor would accept male students: "My friend’s experience in the hospital…they were complaining that they're not able to find research…I told them like there's so many doctors in the hospital, just go to them. And they said…we always try, and we just get rejected, but at the same time there are guys who are working with the professor with the same doctors. So it's kind of weird [that] they don't take them seriously."

Moreover, women also found it difficult to approach male faculty. One participant noted that “your mentor, he might not be open to the idea of training females.” However, other participants reported that they felt students were given equal opportunities. These students noted that doctors placed greater emphasis on the willingness of the student to work. As one participant observed, “I think I largely agree…in the sense that they don't really differentiate between…male students and female students.”

Some of the difficulty arose from faculty members’ assumptions about female students’ research interests. For example, one participant “approached a….surgeon, and I asked him if he had any projects he was…doing that I could take part in,” because, as she told him, “I do want, you know, some experience in surgery research.” The student reported that the surgeon “was surprised” by her request and responded, “most girls usually are not interested in surgery.” The student reported that this exchange motivated her to “pave the way kind of for more girls” and said that it was “all the more reason to put myself in surgical research.”

Another recurring theme was the concept of “shyness,” a term that participants mentioned both directly and indirectly in different interviews. Participants discussed feeling shy due to their own sensitivity, fear of bothering busy faculty, and concern about gender barriers. Some participants reflected on a social barrier that impacted cross-gender communication:

Interviewer: I want to come back to the word you used, which is “shy”...why do…female students feel more shy?

Participant: Because [they] had less experience communicating with other gender…sometimes you're worried you would cross the line…you want to show the other person that you're being respectful and that everything is formal…so sometimes it would cause you to be more shy.

These motifs explore the challenges students face in all phases of approaching a doctor or professor for a research opportunity, from shyness to rejection to fear of being compared to others. Overall, these challenges and unconscious biases create barriers for female students to fully engage in and access research opportunities.

Theme 3: practical challenges for female students in conducting research

The final set of obstacles our participants discussed was the practical challenges of navigating gendered spaces. Participants noted that these practical challenges could, in various ways, limit their ability to engage fully in necessary research activities.

One example of a persistent (though improving) practical challenge was driving. June 2018 was when driver’s licenses first began to be issued to women in the Kingdom of Saudi Arabia. Many women in this context are either new to driving or still do not drive. Some participants noted that researchers may favor accepting male students, who are more likely to drive. As one participant noted: "Sometimes, certain research or certain studies require you to go back and forth between hospitals a lot, or…you're collecting samples of like, serum or blood or something in one place, and you have to transport it to another place. This transport is something that when researchers are looking for participants for a study, they look for males generally, to do this transport."

The reality that more male students than female students can drive is perceived to influence certain research opportunities. Although more women are getting driver’s licenses, this gap will likely remain a significant practical challenge to conducting research for some years to come.

Another set of practical challenges pertains to the gendered division of space. Several participants mentioned the importance of using the local printer’s shop to order materials necessary for showcasing their research in conferences and competitions. However, the printer’s shop was a male-dominated space in which the female students described feeling uncomfortable and even fearful. One participant said “I went to the printer place to print the poster. It was the worst experience; I will never do it again.” The reason for this reaction was that “The place itself, it was a very small room which was full of males.” She said that “you would not feel safe going inside.” Another participant noted that her female colleagues “did not go inside there.” They gestured to the proprietor that they needed assistance and did their ordering from outside the print shop.

All of the factors we described in this section can present challenges to female students who are keen to engage in research. Female students responded in a variety of ways to these perceived challenges, and the discussion section will explore in more depth both the nature of the obstacles women face and what our findings suggest about how these challenges can be overcome.

## Discussion

There are few articles exploring the underlying causes of the limited participation of women in medical research, the nature of the obstacles women may face in conducting research, and the possible ways to overcome these challenges. Therefore, we conducted this study to pinpoint obstacles participants faced, as well as their various reactions toward these experiences. Our hope is that identifying these challenges will also suggest some possible solutions and paths toward progress.

Gender equality is an important consideration worldwide, particularly regarding education, and we found that women described encountering many gender-related obstacles throughout the research process in various regions [7]. Our literature review highlighted several articles discussing women’s attitudes toward research, women’s involvement in research as compared to their male counterparts, and the ways different research facilities around the world are working toward gender equality in research. For example, all European Union member states still face remarkable difficulties related to gender equality in research [17]. In another study, authors reported that certain obstacles can hinder women physicians from participating in research [18].

One of the main challenges female medical students at Alfaisal University described was their perception of differing expectations for female students compared to expectations for male students. Consequently, women felt that they needed to work harder than their male colleagues to be deemed equally competent. One 2008 study discusses the perception that men are more capable in leadership roles, contributing to the well-known “glass ceiling” that excludes women from higher positions [19]. As evident in our results, women were perceived in a similar manner when conducting research studies and were not expected to perform at the same level as their male counterparts.

The source of these differing expectations may be in historically assigned gender roles. As one of our participants observed, female researchers are often opposing historical expectations for what women are supposed to do. This finding is supported by a study conducted in 2011 that studies the assumption that women are “dependent, nurturing, and submissive” whereas men are “strong, action-oriented, and independent” [20]. These assumptions lead to women being the primary caregivers for their families, which takes time and energy from their career advancement [20]. Another study claims that in academic medicine, women are less likely to be researchers and more likely to be clinicians and educators, tasks that have been referred to as “institutional housekeeping” [21]. As expressed in this study as well as in our results, women sometimes find that being a competent and cutting-edge researcher means stepping outside of the dependent/nurturing/traditionally feminine role and into a strong/action-oriented/traditionally masculine role.

Some theories discussing possible reasons behind the “slow progression of women up the academic ladder” have been proposed. One hypothesis was that women took their jobs less seriously than their male colleagues and, hence, were less successful [22]. This hypothesis was proved invalid since one of the objective measures of productivity was the publication of original research in influential journals, as well as being invited by editors to give opinions on the scientific work of others [23]. Indeed, “when stratified by rank and track,” studies have demonstrated “no gender differences in peer-reviewed publications” [21]. There is, however, a discrepancy among specialties in women being the first authors. In Obstetrics and Gynecology and the Journal of Pediatrics, there was a significant increase in the number of women who were first authors. On the other hand, in journals such as the Annals of Surgery, the number of female first authors remained low [23]. These points reject the hypothesis that women are less serious than men in their work [24]. An alternate narrative may be that women are not interested in certain research fields. When one of our participants was met with surprise when she showed interest in surgery research, it suggested an unspoken boundary around what kinds of research are considered suitable for women.

As mentioned in the results section, some participants expressed the need to outperform their male colleagues to prove that they were worthy of the same opportunities. Supporting this finding is a 2019 study about whether the gender gap in research stems from the evaluation of female investigators or from the quality of their research [25]. The study mentions how, over time, women have felt the need to perform to a higher standard than their male counterparts “to receive equivalent recognition” [25,26].

Mentorship programs that focus on research basics could encourage more women to participate in research. An article published in 2019 in Cameroon describes the establishment of a “Mentor-Protégé” program [27]. In this program, female scientists helped junior female researchers overcome obstacles such as entrenched mindsets about traditional gender roles “through skill-building opportunities for scientific writing and participation in scientific gatherings.” Several proteges acknowledged the positive impact that the mentorship program had on their research experience where their training enabled them to be “more confident to apply for research funding, which resulted in them obtaining research grants, fellowships and travel awards” [27]. Our data suggest that women may benefit from mentorship opportunities that focus on building confidence.

Another obstacle was that female students generally preferred to interact with female faculty, which decreased the research opportunities available to them. This barrier was likely due to cultural beliefs that limit interactions between men and women. A study in Jordan in 2020 demonstrated that Muslim women preferred not to have direct interaction with men beyond what was necessary unless they were first-degree relatives [1]. The participants in our study expressed two primary means of dealing with cross-gender interactions. One group shied away from interacting with male professors and/or asked their male colleagues to approach male faculty members. The other group believed it was important for women to overcome hesitations and approach male faculty members themselves. It was found that less previous experience with cross-gender interactions, according to our participants, caused "shyness," which created a barrier to some kinds of interactions in research settings. This reaction stems from female students’ beliefs that they need to maintain a respectful barrier between themselves and male colleagues or mentors.

This study has several limitations. First, this study primarily targeted female undergraduate medical students at a private university in the college of medicine in the Kingdom of Saudi Arabia. This does not consider the male students’ perspective or the faculty's perspective, which would have provided beneficial points of comparison and counterclaim. The study also excluded other categories of researchers, such as post-graduates and students in other colleges at the university, which would have given broader insight into how different fields perceive opportunities for research in Saudi Arabia. Another element that could provide additional insight is identifying female students who had tried to secure research opportunities but were unsuccessful in their attempts. More information about women’s unsuccessful attempts to engage in research could identify more barriers. Additionally, if we had included a longitudinal component, we may have been able to explore the question of whether perceptions of barriers change over time. Finally, future studies might use a quantitative approach to measure the extent to which the themes we discovered are more broadly applicable to undergraduate female medical students in Saudi Arabia and other areas in the Middle East.

## Conclusions

This is the first qualitative study from Saudi Arabia that explores female undergraduate medical students’ perspectives on barriers to conducting research. Despite recent advances, gender imbalance persists in research production in various scientific fields, including medicine, and many obstacles remain in fully engaging women in research activities and creating the conditions in which they can thrive. The key themes found in our study were differing expectations for female students versus male students in conducting research, challenges for female students in securing research opportunities, and practical challenges for female students in conducting research. Given the obstacles faced, we might expect that they would discourage engagement in research; however, the female medical students we interviewed showed commitment to overcoming obstacles in conducting research, as evidenced by their high rate of participation in various research activities. Some of them also expressed a desire to pave the way for more women to do research in the future.

This study also highlights the importance of further exploration of this field. While this study’s research questions focused on female students’ perspectives, future projects could broaden the scope by including male students’ and faculty members’ perspectives on gender issues in medical research in the Middle East. Furthermore, comparative studies could analyze differences across medical colleges in the region and could explore new strategies for engaging female medical students in research.

## Appendices

Interview script

The following is a guideline followed by all interviewers in the process of attaining this research. Kindly note that as this interview followed a semi-structured format, the general questions asked remained constant throughout each interview, and additional questioning was obtained in relation to the flow of the conversation with the participants to obtain comprehensive data.

Introduction

Thank you for agreeing to help us with this project.

The purpose of this interview is to better understand the female students’ perspective on research opportunities in Saudi Arabia.

Allow me to further explain the project: research has been progressing rapidly throughout the years, and females’ contribution has been significantly increasing. Therefore, the female perspective is an important factor to identify some of the possible obstacles that women face when conducting research and eventually encourage more females to engage in research. Despite recent advances, gender imbalance persists in research production in many scientific fields, including medicine. We aim to explore female students’ perspectives toward research and to find obstacles that might contribute to the gender gap in the research field.

The interview is confidential, meaning your name will not be disclosed, but the information you provide to us will be reported in a publication. We will record your response to each individual question to transcribe verbatim for analysis to help ensure that all information is captured accurately. The recording will not be shared with anyone outside this research team.

Should you decide during your interview that you would like to stop recording, you may do so at any time. May I record this interview? Do you have any questions before we begin? Please sign this consent form.

Interview Questions (Open-Ended)

How long have you been a student at Alfaisal University? What is your academic year?

Can you tell us about any research you have been involved in throughout your time at Alfaisal University?

Can you elaborate on your experience while conducting the research?

Can you share any positive or negative experiences while doing research?

Were there any obstacles you faced throughout the course of the project(s)?

In your opinion, do you think that your gender played a role in making it difficult, or easy, to find suitable research opportunities?

Can you give any specific instances which made you realize that gender makes it more difficult/easier to be involved in research?

How was your experience working in research teams?

In your opinion, do you believe your gender played a role in your experience working with the team?

How was your experience with your research mentor/faculty?

In your opinion, do you believe your gender played a role in your experience working with your research mentor/faculty?

In your opinion, what are the barriers (if any) for female students participating in research at Alfaisal University?

Conclusions and Wrap-Up

Before we end this interview, are there any last comments you have regarding this area of research?

Thank you for your participation.

## References

[1] Subeh ZA, Alzoubi K (2020). Cultural and religious barriers influencing the participation of women in research: a study from Jordan. Gender Issues.

[2] Hren D, Lukić IK, Marusić A, Vodopivec I, Vujaklija A, Hrabak M, Marusić M (2004). Teaching research methodology in medical schools: students' attitudes towards and knowledge about science. Med Educ.

[3] Albahari D, Bashir M (2020). Gender gap in mental health research productivity: results from Qatar. Asian J Psychiatr.

[4] Abun D (2021). The attitude of graduate students toward research and their intention to conduct research in the future. Social Science Research Network.

[5] Abu-Zaid A, Alnajjar A (2014). Female second-year undergraduate medical students' attitudes towards research at the College of Medicine, Alfaisal University: a Saudi Arabian perspective. Perspect Med Educ.

[6] Salgueira A, Costa P, Gonçalves M, Magalhães E, Costa MJ (2012). Individual characteristics and student's engagement in scientific research: a cross-sectional study. BMC Med Educ.

[7] Al-Hilali SM, Al-Kahtani E, Zaman B, Khandekar R, Al-Shahri A, Edward DP (2016). Attitudes of Saudi Arabian undergraduate medical students towards health research. Sultan Qaboos Univ Med J.

[8] Kharraz R, Hamadah R, AlFawaz D, Attasi J, Obeidat AS, Alkattan W, Abu-Zaid A (2016). Perceived barriers towards participation in undergraduate research activities among medical students at Alfaisal University-college of medicine: a Saudi Arabian perspective. Med Teach.

[9] Mina S, Mostafa S, Albarqawi HT, Alnajjar A, Obeidat AS, Alkattan W, Abu-Zaid A (2016). Perceived influential factors toward participation in undergraduate research activities among medical students at Alfaisal University-college of medicine: a Saudi Arabian perspective. Med Teach.

[10] Abu-Zaid A, Altinawi B, Eshaq AM (2018). Interest and perceived barriers toward careers in academic medicine among medical students at Alfaisal University - college of medicine: a Saudi Arabian perspective. Med Teach.

[11] Alsaleem SA, Alkhairi MA, Alzahrani MA (2021). Challenges and barriers toward medical research among medical and dental students at King Khalid University, Abha, Kingdom of Saudi Arabia. Front Public Health.

[12] Landman M (2006). Getting quality in qualitative research: a short introduction to feminist methodology and methods. Proc Nutr Soc.

[13] Palinkas LA, Horwitz SM, Green CA, Wisdom JP, Duan N, Hoagwood K (2015). Purposeful sampling for qualitative data collection and analysis in mixed method implementation research. Adm Policy Ment Health.

[14] Clarke V, Braun V (2016). Thematic analysis. J Posit Psychol.

[15] Kiger ME, Varpio L (2020). Thematic analysis of qualitative data: AMEE Guide No. 131. Med Teach.

[16] Gale NK, Heath G, Cameron E, Rashid S, Redwood S (2013). Using the framework method for the analysis of qualitative data in multi-disciplinary health research. BMC Med Res Methodol.

[17] Striebing C, Kalpazidou Schmidt E, Palmén R, Holzinger F, Nagy B (2020). Women underrepresentation in R&I: a sector program assessment of the contribution of gender equality policies in research and innovation. Eval Program Plann.

[18] Rouse LP, Nagy-Agren S, Gebhard RE, Bernstein WK (2020). Women physicians: gender and the medical workplace. J Womens Health (Larchmt).

[19] Carnes M, Morrissey C, Geller SE (2008). Women's health and women's leadership in academic medicine: hitting the same glass ceiling?. J Womens Health (Larchmt).

[20] Zhuge Y, Kaufman J, Simeone DM, Chen H, Velazquez OC (2011). Is there still a glass ceiling for women in academic surgery?. Ann Surg.

[21] Wright AL, Schwindt LA, Bassford TL, Reyna VF, Shisslak CM, St Germain PA, Reed KL (2003). Gender differences in academic advancement: patterns, causes, and potential solutions in one US College of Medicine. Acad Med.

[22] Schroen AT, Brownstein MR, Sheldon GF (2004). Women in academic general surgery. Acad Med.

[23] Jagsi R, Guancial EA, Worobey CC (2006). The "gender gap" in authorship of academic medical literature--a 35-year perspective. N Engl J Med.

[24] Housri N, Cheung MC, Koniaris LG, Zimmers TA (2008). Scientific impact of women in academic surgery. J Surg Res.

[25] Witteman HO, Hendricks M, Straus S, Tannenbaum C (2019). Are gender gaps due to evaluations of the applicant or the science? A natural experiment at a national funding agency. Lancet.

[26] Reuben E, Sapienza P, Zingales L (2014). How stereotypes impair women's careers in science. Proc Natl Acad Sci U S A.

[27] Mekongo PE, Nolna SK, Ngounoue MD (2019). The Mentor-Protégé program in health research in Cameroon. Lancet.

